# Decision Making as a Learned Skill in Mice and Humans

**DOI:** 10.3389/fnins.2022.834701

**Published:** 2022-03-11

**Authors:** Michael I. Posner, Aldis P. Weible, Pascale Voelker, Mary K. Rothbart, Cristopher M. Niell

**Affiliations:** ^1^Institute of Neuroscience, University of Oregon, Eugene, OR, United States; ^2^Department of Psychology, University of Oregon, Eugene, OR, United States; ^3^Department of Biology, University of Oregon, Eugene, OR, United States

**Keywords:** decision, executive attention network, hippocampus, neuromodulation, optogenetics, orienting network

## Abstract

Attention is a necessary component in many forms of human and animal learning. Numerous studies have described how attention and memory interact when confronted with a choice point during skill learning. In both animal and human studies, pathways have been found that connect the executive and orienting networks of attention to the hippocampus. The anterior cingulate cortex, part of the executive attention network, is linked to the hippocampus *via* the nucleus reuniens of the thalamus. The parietal cortex, part of the orienting attention network, accesses the hippocampus *via* the entorhinal cortex. These studies have led to specific predictions concerning the functional role of each pathway in connecting the cortex to the hippocampus. Here, we review some of the predictions arising from these studies. We then discuss potential methods for manipulating the two pathways and assessing the directionality of their functional connection using viral expression techniques in mice. New studies may allow testing of a behavioral model specifying how the two pathways work together during skill learning.

## Introduction

One example of decision making is reaching a fork in a path, requiring the choice of one path in order to obtain a goal or avoid an unpleasant outcome (Hastie and Dawes, 2010). This characterization views rational human decision making as a choice point. It also fits for a rodent that moves left rather than right in a maze, a monkey that remains fixated when a peripheral cue is flashed, and a human pressing a key when two letters are identical. Decisions made at these choice points are influenced by both task-specific learning and innate biases. Making decisions can be viewed as a skill like much of thinking (Bartlett, 1958).

Learning a skill involves an interaction between attention and memory (Recanzone et al., 1992; Chun and Turk-Browne, 2007; Weible, 2013; Panichello and Buschmann, 2021). Our goal here is to use what we know about attention and memory networks to outline the neural basis of the skill of decision making. In pursuit of this goal, we first examine the relationship between attention and memory networks in rodents, monkeys, and humans. Drawing upon the literature and our own studies we outline two pathways by which attention and memory networks interact during skill learning. We then consider the function of each pathway during the various stages of skill learning. Finally, we point out gaps in the literature and methods that might allow us to fill those gaps.

## Humans and Other Animals

**FIGURE 1:**
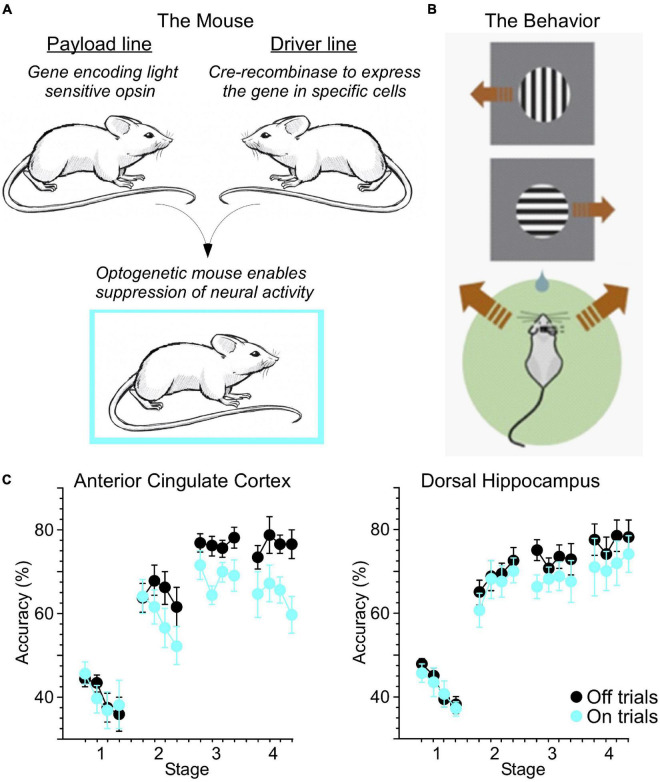
Optogenetic mouse models enable new ways to test skill learning. **(A)** Breeding together of payload and driver lines enabled cell-type specific expression of light-sensitive opsins. **(B)** Water-restricted, head-fixed mice were trained to run left or right on a ball in response to visual cues at the top or bottom (respectively) of a computer screen for a water reward. Brown arrows indicate running direction. **(C)** Light was delivered through chronically implanted optic fibers to the anterior cingulate cortex (ACC) or dorsal hippocampus (dHC) at the initiation of 20% of trials (On trials). Suppression of ACC activity reduced accuracy across multiple stages of training. Suppression of dHC more selectively impacted performance late in training (adapted from Weible et al., 2019).

It is reasonable to begin with a simple question: to what degree is skill learning similar between humans and other animals? In human skill learning, changes in performance and reaction time have been described as fitting a power function (Fitts and Posner, 1967; Anderson, 1982). Similarly, we found that in mice trained in a 2-alternative forced choice (2-AFC) task, reaction time decreased and accuracy increased roughly as a power function relative to training time (Weible et al., 2019). In our study, we utilized optogenetics to enable suppression of activity in excitatory neurons through activation of inhibitory parvalbumin-positive interneurons (PV-INs). This approach involves breeding together two lines of mice, a “payload” line and a “driver” line (Figure 1A). The payload line carries the gene sequence encoding the light sensitive opsin (in this case, the excitatory channelrhodopsin-2 or ChR2). However, expression of this payload gene is dependent on Cre-recombinase, which is expressed in specific cell types (in this case, the PV-INs) in the driver line. Mice bred from these two lines thereby express ChR2 in PV-INs. Delivery of blue (445 nm) light to the brain *via* chronically implanted optic fibers stimulates these PV-INs, resulting in overall suppression of excitatory activity.

Mice were held in place *via* a metal headplate, and trained to run in place atop a Styrofoam ball able to spin freely on a cushion of air (Figure 1B). The mice faced a computer monitor presenting visual cues, and a water reward was delivered when the mouse “ran” in the correct direction for the corresponding cue (in this case, rotating the ball to the left for a cue presented at the top of the screen, and right for a cue at the bottom). Using this approach, we suppressed neural activity as mice trained, in two key areas involved in learning: the hippocampus (HC), usually thought to be involved in forming associations, and the anterior cingulate cortex (ACC), a major node of the executive attention network (Petersen and Posner, 2012). Optogenetic suppression of these areas reduced accuracy during different stages of learning (Figure 1C).

There is also evidence that both rodents and humans orient their attention to a cue when a subsequent target is more likely to occur at the location of the cue (valid) than other locations (invalid) (Beane and Marrocco, 2004). The size of the validity (orienting) effect is similar in rats, monkeys, and humans. Studies also found similar findings for the orienting network of mice (Wang and Krauzlis, 2018; Li et al., 2021), which is the species used in our study of learning described above. When a double cue indicates that a target will occur soon (alerting), but not where it is located, the improvement in reaction time due to the alerting effect is also similar in the rats, monkeys, and humans (Beane and Marrocco, 2004). However, overall, reaction times are faster in humans and slower in monkeys but this may reflect differences in training and in the details of the stimulus presentation and response recording.

There is also evidence that all three species have a frontally based executive system that is active during learning and when there is conflict between responses (Washburn, 1994; Weible, 2013), but this network is likely much more developed in humans.

## Connecting Attention and Memory Networks

In the human, the ACC and adjacent medial prefrontal cortex (mPFC) are key nodes mediating the attention needed to resolve conflict (Botvinick et al., 2001; Petersen and Posner, 2012). In the 2-AFC task described above, mice are required to execute a movement that is orthogonal to the axis in which the stimuli are presented. This introduces a conflict which must be overcome before the correct decision can be made. It takes weeks of training for mice to reach our 80% correct criterion. Mouse learning like human learning showed a power function between time of training and response accuracy. When we suppressed output from the mouse ACC using optogenetics, accuracy in the visual 2-AFC task was significantly reduced across all stages of training, potentially revealing the inability to resolve this conflict during suppressed trials (see Figure 1C, left). Suppression of HC output reduced accuracy in the middle and late stages of learning (see Figure 1C, right; Weible et al., 2019). In addition, in both rodents (Akam et al., 2021; Tervo et al., 2021) and in humans (Petersen and Posner, 2012) the ACC seems to play an important role in executive control and behavioral predictions. Despite these similarities we recognize that functions of the mouse ACC need more investigation.

**FIGURE 2:**
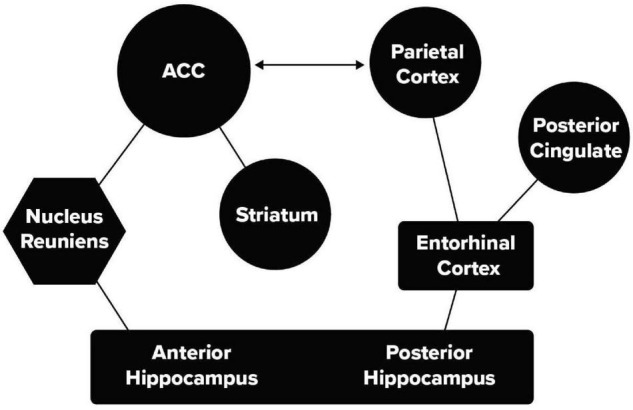
Two pathways between the memory and attention networks. A thalamic pathway including HC, nucleus reuniens of the thalamus, and the ACC and a second pathway including HC, the entorhinal cortex and parietal lobe.

Recent mouse and human studies have identified two pathways that can connect the HC and ACC. These are illustrated in Figure 2. This figure distinguishes between the anterior and posterior HC used in human studies, in mice these correspond to the ventral (anterior) and dorsal (posterior) HC.

While the two pathways may overlap somewhat, they have been identified with distinct functions. One mouse study (Xu and Südhof, 2013) showed that generalization of learned fear depended on a pathway from the ventral, HC through the nucleus reuniens of the thalamus (nRe) to the mPFC (McKenna and Vertes, 2004). When this pathway (Figure 2) was suppressed, fear was elicited only in the location of learning and did not generalize to other cages. In a human study of category learning (Bowman and Zeithamova, 2018) stimuli were used that were distortions of one of four prototype figures. Following learning to make the same response to the four distortions of a given prototype, it was found that the unshown prototype was stored in the anterior portion of the hippocampus in interaction with the ventral mPFC (Bowman and Zeithamova, 2018).

Human evidence is not limited to generalization. In the think/no-think task, subjects are trained on an association between a stimulus and response. After training they are shown the stimulus with the instruction either to think of the response or to inhibit thought (Anderson et al., 2004). On trials suppressing thought of the response, stronger activity in the ACC was accompanied by reduced activity in the HC. In rats the pathway between ventral mPFC and HC has been shown to be involved generally in inhibitory control (Chudasama et al., 2012). Reciprocal connectivity links the mPFC and the ACC (Jones et al., 2005). Thus, connections between ACC and adjacent mPFC and HC are involved in both control of memory by attention and generalization.

The role of a posterior medial pathway in episodic memory has been well described in a recent review (Ritchey and Cooper, 2020). An important finding from the think/no-think task relates to the connection from the HC through the entorhinal cortex (Figure 2) to the posterior parietal lobe involved in the orienting of attention. On those trials in which the participants were instructed not to think of the response, but reported to the experimenter that they did in fact think about it, MRI data showed activity in the posterior parietal cortex (Levy, 2008; Anderson et al., 2016). The same area of the parietal cortex was active when attempting to suppress the response irrespective of content (Anderson et al., 2016). A study by Levy (2008) using a cuing task to move the persons’ orienting to the target location covertly, without causing eye movement, showed activity in a part of the parietal lobe that clearly overlapped with activity observed when retrieving a suppressed association. This suggests that suppression of retrieval from memory uses a second route through an attentional network involving the parietal lobe that is also used in orienting attention to an external stimulus. One view (Anderson et al., 2016) is that the anterior network is involved in suppression proactively while the posterior pathway is more important for suppression during retrieval.

Most rodent studies of the HC have stressed its involvement in spatial navigation, as originally described by O’Keefe and Nadel (1978; also O’Keefe and Dostrovsky, 1971). Most human studies stress a broader role in associative learning regardless of content. A monkey study provides an important perspective on the possible evolution of this network. In their study, Wilming et al. (2018) demonstrated that covert orienting modifies function in the entorhinal cortex, thus illustrating that spatial attention is central to the function of the pathway between HC and the cortex. Another rodent study used a 5-choice reaction time task that required the rat to orient to the location of the target. The findings in this task suggest attentional deficits from either peri- or postrhinal lesions (Trettel et al., 2021). From performance in a set of post-learning tasks, the authors argue that the perirhinal cortex involves more endogenous attention, while the postrhinal area involves stimulus driven attention. This latter point is somewhat confounded by overall differences in the learning of the original task, but it is consistent with the entorhinal pathway being heavily involved in attention to spatial location.

Evidence that the failed effort to suppress awareness of a learned response regardless of content activates the orienting-of-attention network (Anderson et al., 2016) supports a central role of spatial location during memory retrieval. The pathway may mostly involve spatial navigation in rodents, but evolves into a more general retrieval role in primates and humans. The role of the orienting pathway suggests that knowing where a memory occurred can be an important aid to retrieval. Perhaps the importance of spatial location in this pathway underlies the strength of the use of the method of loci to improve memory for stored events (Bower, 1970). Additional tests of the evolution of this pathway could be useful in further understanding its function in humans.

## Function of Pathways

The work summarized so far involves studies of animals and task-related imaging in humans. It indicates that the anterior pathway between HC and ACC through the thalamus is important in generalization and cognitive control of the hippocampus, perhaps improving storage of attended items and inhibiting ones less important. The posterior pathway linking the HC and the parietal lobe *via* entorhinal cortex represents the evolutionarily old pathway for navigation in rodents and serves as the source of spontaneous retrieval of unwanted thoughts regardless of content in humans. The rodent work summarized in this article is limited to the relatively simple incompatible reaction time task we used (Weible et al., 2019). This limitation is reduced somewhat by the similar conclusion from more complex human studies involving categorization or the think no-think procedure. While these methods may not be useful for mouse study, there has been a recent growth in the use of naturalistic behaviors for the mouse (Juavinett et al., 2018), including prey capture, which involves elements of detection and orienting (Hoy et al., 2019). This work may allow models of more realistic rodent behavior to emerge.

A recent study of HC-cortical connections reveals both anterior and posterior pathways from human HC to cortex similar to those described in Figure 2 (Zheng et al., 2021). This study involves resting state MRI. The anterior pathway goes directly to the mPFC and is shown to be related to the default state (Raichle et al., 2001), which the authors argue is important in self-relevant rumination. The posterior network involving the entorhinal cortex and parietal lobe is involved in storage and retrieval of many types of memories. These studies do not discuss the ACC directly but describe the adjacent mPFC as related to the default network. The evidence for resting state self-reflection in humans is reasonable, but seems somewhat less compelling in rodents.

This resting state study does not take into account the task-relevant work on modification of the HC by the ACC and the involvement in generalization discussed earlier in this article. While the two pathways shown in Figure 2 and those discussed by Zheng et al. (2021) are anatomically similar and are involved in functions related to memory retrieval, the details in their function are somewhat different. The work discussed earlier in this article shows that the thalamic pathway is involved in generalization and cognitive control of memory and is not unique to self-reflection. While we argue that the posterior pathway has a unique relation to sensory orienting, Zheng et al. (2021) do not discuss the special role of the parietal lobe in this function. These differences in pathway function should be fruitful for designing studies to distinguish the differences in the functional roles for the connections between attention and memory.

Critical to the design of new studies is the ability to determine the direction of information flow. Flow from the ACC, mPFC or parietal lobe may indicate top-down control of the HC regarding storage or preventing interfering information during retrieval. Bottom-up flow from the HC to cortex may be involved in retrieval and consolidation as information is distributed to other relevant networks. While distinguishing the direction of influence is difficult, we discuss one promising method in the next section.

## Neuromodulation

In 2010 we reported that 2–4 weeks of meditation training changed connectivity of the white matter surrounding the ACC as measured by fractional anisotropy in diffusion tensor imaging (Tang et al., 2010, 2012). To understand the mechanisms involved in this finding we used optogenetics to rhythmically manipulate neuronal activity in the mouse ACC (Piscopo et al., 2018). We stimulated the ACC at 1, 8, or 40 Hz in a half-hour session for 20 days and compared the effects on myelination to unstimulated controls. We found significant changes in the g-ratio (axon diameter/axon + myelin diameter) in the corpus callosum following rhythmic stimulation at 1 and 8 Hz but not at 40 Hz. Since frontal theta is increased following meditation training (Xue et al., 2014), we attempted a 20-day theta stimulation regimen in humans using scalp electrodes targeted at the ACC while they performed a task that also recruited the ACC. This effort did change the level of theta activity in the ACC more than in non-targeted areas, but did not alter white matter (Voelker et al., 2021). We think it likely that theta may first change synaptic activity, followed over a longer timescale by a change in white matter. In support of this idea is that even brief rhythmic theta stimulation has been shown to improve attention (Reinhart, 2017) and memory (Roberts et al., 2018). A possible mechanism might be enhanced Long Term Potentiation (LTP) when accompanied by theta stimulation (Larson and Munkacsy, 2015). Thus rhythmic theta stimulation might serve to improve connectivity by enhancing synaptic activation.

Our current goals are to better understand whether targeted stimulation in mice can improve connectivity of the two pathways shown in Figure 2. One problem is that for subcortical connections it is difficult to determine the direction of influence. While cortical areas such as the parietal lobe and prefrontal cortex segregate cells in different lamina, this segregation is less evident in subcortical areas. Thus, it is hard to distinguish between memory-to-attention and attention-to-memory control. One possible approach is to use a 2-virus method in mice, to isolate and separately manipulate top-down and bottom-up pathways of information flow (Gore et al., 2013). For example, to control the bottom-up thalamic route through nRe one would inject the ACC with one virus that retrogradely transports Cre-recombinase back to ACC projection neurons in nRe, and then inject a second virus in nRe carrying a Cre-dependent version of a light-gated opsin that enables manipulation of neural activity. Because only ACC-projecting cells in nRe have Cre-recombinase (through retrograde delivery by the first virus), only those cells will express the Cre-dependent opsin (delivered by the second virus). Thus, light delivered through surgically implanted optic fibers in nRe will enable the suppression or stimulation of those cells, and only those cells, projecting to ACC. The same approach can be employed to target ACC neurons in the top-down projection. Cells projecting from HC to parietal lobe through the entorhinal cortex can also be examined by a similar strategy allowing facilitation or suppression of each direction of the pathway. Specific tasks can be designed in the mouse allowing suppression of the ACC and HC by optogenetics to examine the function of the thalamic and entorhinal cortex pathways.

Another advantage of this approach is that the direction of information may be measured at each stage of learning. Suppose, for example, that early stages of learning show the thalamic pathway to be involved in control of activity in the HC, but later stages show it to be involved in generalization. If the direction of activity is from ACC to HC this would be consistent with early cognitive control, while the direction HC to ACC, later in learning, might be involved in distribution of information to different brain areas in the process of consolidation. The thalamic pathway being mainly top-down early in learning and bottom-up late in learning would be consistent with an early cognitive stage and a late stage more related to consolidating and generalizing learned content (Fitts and Posner, 1967).

If this work is successful, we can then rhythmically stimulate each pathway in each direction in the theta band and determine which functions are changed and in what direction. These findings may provide a better understanding of how learning shapes decisions concerning the response. The findings may not be limited to spatial decisions but may apply more generally to all forms of learned decision making.

## Author Contributions

AW conducted the experiment in Figure 1 under direction of CN. MP and MR developed the model in Figure 2. All authors reviewed the literature and contributed to writing.

## Conflict of Interest

The authors declare that the research was conducted in the absence of any commercial or financial relationships that could be construed as a potential conflict of interest.

## Publisher’s Note

All claims expressed in this article are solely those of the authors and do not necessarily represent those of their affiliated organizations, or those of the publisher, the editors and the reviewers. Any product that may be evaluated in this article, or claim that may be made by its manufacturer, is not guaranteed or endorsed by the publisher.
